# Persistence of *Mycoplasma genitalium* Following Azithromycin Therapy

**DOI:** 10.1371/journal.pone.0003618

**Published:** 2008-11-03

**Authors:** Catriona S. Bradshaw, Marcus Y. Chen, Christopher K. Fairley

**Affiliations:** 1 Department of Epidemiology and Preventive Medicine, Monash University, Melbourne, Victoria, Australia; 2 Melbourne Sexual Health Centre, The Alfred Hospital, Melbourne, Victoria, Australia; 3 School of Population Health, University of Melbourne, Melbourne, Victoria, Australia; Tulane University, United States of America

## Abstract

**Background:**

To determine clinical outcomes and cure rates for *M.genitalium* genital infection in men and women following azithromycin 1 g.

**Methodology:**

Patients attending Melbourne Sexual Health Centre between March 2005 and November 2007 with urethritis/epididymitis, cervicitis/pelvic inflammatory disease and sexual contacts of *M.genitalium* were tested for *M.genitalium* by polymerase chain reaction (PCR). *M.genitalium*-infection was treated with 1 g of azithromycin and a test-of-cure (toc) was performed one month post-azithromycin. Response to azithromycin, and response to moxifloxacin (400 mg daily for 10 days) in individuals with persistent infection post-azithromycin, was determined.

**Principal Findings:**

Of 1538 males and 313 females tested, 161 males (11%) and 30 females (10%) were infected with *M.genitalium*. A toc was available on 131 (69%) infected individuals (median = 36 days [range 12-373]). Of 120 individuals prescribed azithromycin only pre-toc, *M.genitalium* was eradicated in 101 (84%, 95% confidence intervals [CI]: 77–90%) and persisted in 19 (16%, 95% CI: 10–23%). Eleven individuals with persistent infection (9%, 95% CI: 5–15%) had no risk of reinfection from untreated-partners, while eight (7%, 95% CI: 3–12%) may have been at risk of reinfection from doxycycline-treated or untreated-partners. Moxifloxacin was effective in eradicating persistent infection in all cases not responding to azithromycin. Patients with persistent-*M.genitalium* were more likely to experience persistent symptoms (91%), compared to patients in whom *M.genitalium* was eradicated (17%), p<0.0001.

**Conclusion:**

Use of azithromycin 1 g in *M.genitalium*-infected patients was associated with unacceptable rates of persistent infection, which was eradicated with moxifloxacin. These findings highlight the importance of follow-up in *M.genitalium*-infected patients prescribed azithromycin, and the need to monitor for the development of resistance. Research to determine optimal first and second-line therapeutic agents for *M.genitalium* is needed.

## Introduction

*M.genitalium* is a cause of urethritis in men and cervicitis in women [1], has been isolated from women with endometritis and salpingitis, and has been implicated in tubal factor infertility [2], [3], [4], [5], [6]. *In vitro* studies have shown that *M.genitalium* is most susceptible to macrolides, particularly to azithromycin, but that it has reduced susceptibility to tetracyclines and some fluoroquinolones [7], [8]. Clinical studies using doxycycline and levofloxacin also show significant failure rates following these therapies [8], [9], [10], [11], [12], [13]. Two trials have compared azithromycin to doxycycline for the treatment of *M. genitalium*. In both of these, 1 g of azithromycin was shown to be more effective (84–85%) than 7–9 days of doxycyline (35–36%) [12], [13]. In 53 individuals failing doxycycline, an extended course of azithromycin (500 mg followed by 250 mg daily for 4 days) eradicated *M. genitalium* in 47 men (96%) and 6 (100%) women. However, extended azithromycin therapy was not statistically superior to azithromycin 1 g [12].

Current international guidelines recommend azithromycin 1 g as first-line therapy for uncomplicated *M.genitalium* infection [14]; however second-line agents have not been extensively evaluated. Importantly, azithromycin 1 g is also first-line therapy for non-gonococcal urethritis (NGU) in most countries, resulting in a high proportion of patients with symptomatic *M.genitalium*-positive NGU receiving azithromycin presumptively. In 2006 we reported high rates of treatment failure following azithromycin 1 g in men with *M.genitalium*-associated urethritis (28%, 95% confidence interval (CI),15–45%) [15]. Reduced susceptibility to azithromycin of these isolates demonstrated that azithromycin-resistance rather than re-infection was responsible for treatment failure, and that test positivity was not due to detection of persistent non-viable DNA. *M. genitalium* was successfully eradicated in all men experiencing azithromycin failure using 400 mg daily of moxifloxacin for 10 days.

In this study, we aimed to determine likely levels of azithromycin treatment failure using a large number of *M. genitalium*-infected individuals. Furthermore, we sought to determine the clinical characteristics of individuals infected with *M.genitalium*, including those who failed to respond to azithromycin, and to examine treatment response to moxifloxacin in those in whom azithromycin did not eradicate infection. Establishing levels of azithromycin resistance and effective second-line agents for the treatment of *M.genitalium* is of considerable clinical and public health importance to the field.

## Methods

This study was undertaken at the Melbourne Sexual Health Centre (MSHC) between March 2005 and November 2007. During that period, routine testing for *M.genitalium* was undertaken for urethritis, epididymitis, cervicitis, pelvic inflammatory disease (PID) and for sexual contacts of *M.genitalium*-infected men and women. First pass urine samples or urethral swabs were recommended for testing for *M.genitalium* in males, and cervical swabs were recommended for testing in females, unless clinician or patient preference necessitated high vaginal or first pass urine samples to be obtained from females. Standardized epidemiological and clinical data were collected electronically at the time testing for *M.genitalium* was undertaken.

*M.genitalium*-infected men and women were treated with azithromycin 1 g. All patients were asked to notify sexual partner(s) to obtain testing and treatment. A routine test-of-cure (toc) was recommended one month following azithromycin. Clinic nurses contacted patients fortnightly on up to three occasions if they had not attended for the toc. In those testing positive 3 weeks or more following treatment, and where reinfection was considered unlikely, moxifloxacin 400 mg daily for 10 days was administered, with another toc one month after treatment with moxifloxacin.

Male contacts of men with *M.genitalium* were screened for *M.genitalium* using throat, rectal and first-void urine samples. Female contacts were tested using cervical, vaginal or first-void urine samples. Contacts received presumptive treatment with azithromycin 1 g, unless their partner had failed azithromycin therapy in the absence of reinfection. In such cases, moxifloxacin was administered. Testing for *M.genitalium* was by polymerase chain reaction (PCR) according to the method described by Yoshida et al. [16].

Data on the number of patients tested for *M.genitalium*, the proportions testing positive, and demographic and behavioural characteristics were analysed. Case files were reviewed in individuals who tested positive for *M.genitalium* to establish the treatment(s) prescribed to the index and sexual contacts, and the clinical outcomes following treatment. Data were obtained to determine the clinical characteristics of individuals in whom *M.genitalium* persisted, the likelihood of reinfection from untreated partners, and the response to treatment with moxifloxacin in those failing to respond to azithromycin. Treatment outcomes following azithromycin were recorded as eradication of *M.genitalium* if it was not detected at toc1, or persistent infection with or without risk of reinfection from sexual partners. All individuals in this study had attended for medical care at MSHC. In line with the policy of our institutional ethics committee and guidelines of the National Health and Medical Research Council of Australia, consent and ethics approval were not required, as this was an analysis of routinely collected clinical data. Data were analysed using SPSS (Version 15, Chicago, USA). Ninety-five percent confidence intervals (CIs) were calculated for proportions. Differences between categorical variables were compared using the Chi-square or Fisher's Exact test where appropriate. Patients were excluded from the analysis where information or specimens were not available.

## Results

During the study period, 1538 men and 313 women were tested for *M.genitalium*: 191 (10%) tested positive for *M.genitalium* (161 males [11%] and 30 females [10%]), 1660 (89%) patients tested negative, and 5 (0.2%) had specimens that were repeatedly unassessable. There were no significant differences in demographic, behavioural or clinical characteristics between infected and uninfected men and women other than infected individuals were more likely than uninfected to identify as a contact of *M.genitalium* (data not shown), p<0.01.

### Clinical and laboratory characteristics of M.genitalium-infected patients

Table 1 shows the clinical characteristics of *M.genitalium*-infected men and women, the specimens used to detect *M.genitalium*, and the presence of genital coinfections. The median duration of symptoms prior to presentation for males and females was 7 days (range 1–120) and 10 days (range 1–360) respectively. Urethral smears for Gram stain were obtained on 139 (86%) men with urethral *M.genitalium*; 89 (64%) had ≥5 polymorphonuclear cells (PMNs) per high power field on urethral smear. Vaginal and cervical PMN counts were only available on 19 (60%) and 16 (57%) women respectively; of these, 10 (52%) had ≥5 PMN per high power field on vaginal smear and 10 (63%) had ≥5 PMN per high power field on cervical smear.

**Table 1:** Clinical and Laboratory Characteristics of Participants Infected with *M.genitalium*.

	Male n = 161 (%)	Female n = 30 (%)
***Specimens in which M.genitalium was detected***		
**First pass urine**	**155 (96)**	**3 (10)**
**Urethral swab**	**2 (1)**	**-**
**Rectal swab**	**4 (3)**	**-**
**Cervical swab**	**-**	**23 (77)**
**High vaginal swab**	**-**	**4 (13)**
***Clinical characteristics***		
**Urethral discharge**		
**no**	35 (22)	
**yes**	126 (78)	
**Dysuria**		
**no**	71 (44)	-
**yes**	90 (56)	5 (17)
**Urethral Irritation/itch**		
**no**	116 (72)	
**yes**	45 (28)	
**Meatal Inflammation**		
**no**	138 (86)	
**yes**	23 (14)	
**Vaginal Discharge**		
**no**	-	15 (50)
**yes**	-	15 (50)
**Dyspareunia**		
**no**	-	25 (83)
**yes**	-	5 (17)
**Lower Abdominal Pain**		
**no**	-	23 (77)
**yes**	-	7 (23)
**Abnormal vaginal bleeding**		
**no**	-	25 (83)
**yes**	-	5 (17)
**Mucopurulent cervicitis**		
**no**	-	23 (77)
**yes**	-	7 (23)
**Cervical contact bleeding**		
**no**	-	25 (83)
**yes**	-	5 (17)
**Cervical or adnexal tenderness**		
**no**	-	21 (70)
**yes**	-	9 (30)
***Genital coinfections with M.genitalium***		
	***Urethral M.genitalium*** ** (n = 157)** *	***Cervicovaginal M.genitalium*** ** (n = 30)**
***C.trachomatis*** ** (urethra/urine)**		
**Not detected**	141 (90)	-
**Detected**	15 (10)	-
***C.trachomatis*** ** (rectal)#**		
**Not detected**	24 (92)	-
**Detected**	2 (8)	-
***C.trachomatis*** ** (cervix/vagina)**		
**Not detected**	-	29 (97)
**Detected**	-	1 (3)
***N.gonorrhoeae*** ** (urethra)**		
**Not detected**	141 (91)	-
**Detected**	14 (9)	-
***N.gonorrhoeae*** ** (rectal)**		
**Not detected**	26 (93)	-
**Detected**	2 (7)	-
***N.gonorrhoeae*** ** (throat)**		
**Not detected**	27 (93)	-
**Detected**	2 (7)	-
**Bacterial vaginosis**		
**Not detected**	-	15 (56)
**Detected**		12 (44)

* There were no rectal coinfections. #Note rectal and pharyngeal STI screening indicated in MSM (n = 48) only and not performed in all cases. Testing for *Chlamydia trachomatis* was by strand-displacement-amplification (ProbeTec-ETCT-Amplified DNA-Assay, Becton Dickinson, MD, USA). Culture for *Neisseria gonorrhoeae* was performed using modified-Thayer-Martin medium. Vaginal smears assessed for bacterial vaginosis according to the Nugent method.

### Treatment outcomes following azithromycin

A toc was available for 131 (69%) *M.genitalium*-infected individuals after treatment (110 males and 21 females). The median time to returning for the first toc (toc1) was 36 days (range 12–373); 119 (91%) patients returned within 12 weeks of treatment. One hundred and twenty (92%) of these patients had been treated with azithromycin 1 g only prior to toc. Eleven participants (8%), however, were also treated with moxifloxacin for persistent urethral symptoms in the interval following azithromycin but prior to toc1.

Of the 120 patients (102 males and 18 females) treated with azithromycin 1 g who proceeded to toc1, 101 (84%, 95%CI: 77–90%) did not have persistence of *M.genitalium*. In 19 (16%, 95% CI: 10–23%) patients treated with azithromycin, *M.genitalium* persisted at toc1; 11 (9%, 95% CI: 5–15%) were considered by clinicians to not be at risk of reinfection (no sex since treatment or sex with a partner who had been concurrently treated with azithromycin 1 g), Table 2. Eight (7%, 95% CI: 3–12%) patients with a positive toc1 were considered as possibly at risk of reinfection from an untreated or doxycyline-treated partner. Reinfection risk was not consistently documented by clinicians for patients with negative toc1s, so while it would have been helpful to compare reinfection risk in those cured and not cured of *M.genitalium*-infection with azithromycin 1 g, these data were not available.

**Table 2:** Reinfection risk and response of *M.genitalium* to azithromycin at first test of cure (n = 131).

Reinfection risk in patients at toc1	*M.genitalium* detected after 1 g azithromycin at toc1 (n = 19)	*M.genitalium* not detected after 1 g azithromycin at toc1 (n = 101)	*M.genitalium* not detected at toc1 but given moxifloxacin prior to toc1* (n = 11)
**No sex prior to toc1**	4	N/A	9
**Sex prior to toc1 but with a partner concurrently treated with azithromycin**	7	N/A	1
**Possible sex with untreated or doxycycline treated partner prior to toc1**	8	N/A	1
**Reinfection risk not able to be assessed**	0	101	0

toc1 = first test of cure, N/A = data not available (no consistent documentation by clinicians of risk of reinfection in those with negative tests of cure).

* reasons for moxifloxacin use prior to toc1 indicated in the text but all related to persistent symptoms following azithromycin and/or suspected azithromycin resistance.

*M.genitalium* was not detected at toc1 in the additional 11 (8%, 95% CI: 5–14%) patients who received moxifloxacin post-azithromycin but prior to toc1, Table 2. They had been given moxifloxacin at prior to toc1 as they had either already received azithromycin from a physician prior to their presentation but were still symptomatic and tested positive for *M.genitalium* upon attending MSHC (n = 4), or had received azithromycin at presentation to MSHC (n = 7) but had persistent symptoms and were given moxifloxacin prior to toc1. Of the individuals given moxifloxacin pre-toc1 only one had had contact with an untreated partner and may have been at risk of reinfection, the remainder had been celibate or had only had sex with an azithromycin-treated partner.

Data on symptoms at toc1 were available on 119 (91%) patients. Patients with *M.genitalium* detected at toc1 were significantly more likely to report persistence of symptoms (91%), compared to patients in whom *M.genitalium* was not detected (17%), p<0.0001. There was no difference in the proportion of males (23%) compared to females (24%) with persistence of *M.genitalium* post-azithromycin or requiring moxifloxacin prior to toc1 for persistent symptoms. However, heterosexual males were more likely to experience azithromycin failure or to require moxifloxacin for persistent symptoms prior to toc1 (28%) compared to men who have sex with men (MSM),11%, p = 0.04. Sex with a partner from overseas (predominantly Asia) was reported in 20% of patients failing azithromycin or requiring moxifloxacin prior to toc1, compared to only 9% of patients in whom azithromycin eradicated infection, however, this was not statistically significant, p = 0.11. Heterosexual males (19%) were not more likely than MSM (15%) to have had a partner from overseas.

**Figure 1:**
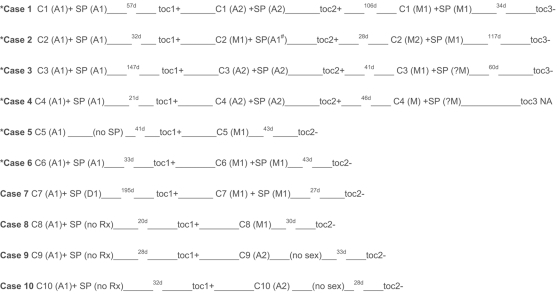
* = cases with no risk of reinfection from untreated sexual partner, d = days, C1 = case 1, C2 = case 2, (A1) = first occasion 1 g azithromycin administered, (A2) = second occasion 1 g azithromycin administered, (M1) = first occasion 400 mg daily of moxifloxacin for 10 days administered, (D1) = first occasion 100 mg bd doxycycline for 7 days administered, SP = sexual partner, no Rx = no treatment administered, toc1 = first test of cure for case, toc2 = second test of cure for case, + = positive, − = negative, NA =  not available, ^#^sp not retreated with azithromycin after first occasion, ? = treatment advised but could not be verified.

Of the 19 individuals with *M.genitalium* detected at toc1, 10 provided a second toc (toc2), Figure 1. Six patients with persistent *M.genitalium*-infection had no risk of reinfection from an untreated sexual partner; 5 had sexual partners who been treated concurrently with azithromycin on one or more occasions, and one had remained celibate. Notably, in three couples simultaneous administration of azithromycin to sexual partners on two consecutive occasions failed to eradicate *M.genitalium*, and it was only when both index and partner were concurrently treated with moxifloxacin did a third toc show that *M.genitalium* had been eradicated. Four patients with persistent infection post-azithromycin may have been at risk of reinfection from an untreated or doxycyline treated partner. Importantly, moxifloxacin eradicated persistent infection following azithromycin in all cases.

## Discussion

This large series provides strong evidence that azithromycin fails to clear *M.genitalium* in a considerable proportion of infected men and women. In only 84% (95% CI: 77–90%) of infected cases treated with azithromycin 1 g was *M.genitalium* eradicated, while infection persisted in 16% of cases; 9% of whom had no risk of reinfection from untreated partners. In an additional 11 (8%) cases use of moxifloxacin preceding toc prevented assessment of azithromycin efficacy; however, moxifloxacin had been prescribed by clinicians for reasons related to probable azithromycin-resistance in the index or their partner. Importantly, moxifloxacin 400 mg daily for 10 days was effective in eradicating persistent infection in all cases in this series who failed to respond to azithromycin, providing further data to support the efficacy of this agent in persistent infection. There was no difference in persistence of *M.genitalium* following azithromycin between males and females; however, heterosexual males, were more likely than MSM to experience persistence of *M.genitalium* following azithromycin therapy.

We previously reported an azithromycin-failure rate of 28% (95% CI: 15–45%) in men with *M.genitalium* urethritis [15]. The numbers in this study were small (n = 32) and the confidence intervals were wide, but reinfection risk was carefully assessed, and no patients had been re-infected or prescribed moxifloxacin prior to a toc being obtained. Antimicrobial susceptibility testing of *M.genitalium* isolates showed increased mean inhibitory concentrations (MICs) to azithromycin >16 mg/l and susceptibility to moxifloxacin (MIC range 0.031–0.125 mg/l). Mutations in region V of the 23S rRNA gene were identified and explained the mechanism of resistance [15]. In this current study we report an azithromycin-failure rate of 16% (95%CI: 10–23%), which may be conservative as it excludes the additional 8% of cases who clinicians felt were experiencing azithromycin-failure and who were treated with moxifloxacin prior to obtaining a toc. While the failure rates differ somewhat in the two series, the confidence intervals overlap and are considerably narrower in the larger series. Most importantly, current clinical expectations are that therapeutic agents for STIs should not be less than 95% effective. Our findings support those of published studies that indicate 1 g of azithromycin at best eradicates *M.genitalium* in only 84–85% of treated patients [12], [13].

Detection of *M.genitalium* by PCR in the early days following therapy may reflect detection of non-viable DNA, and the interval at which to perform a toc for *M.genitalium* following therapy has not been well established. Studies indicate that detection of non-viable *C.trachomatis* DNA following treatment falls over 1–2 weeks [17]; however there are no data available for *M.genitalium*. A significantly higher proportion of cases with persistent detection of *M.genitalium* DNA experienced persistent symptoms (91%) compared to those with negative tocs (17%), indicating, as was found in our previous research, that a positive toc is likely to reflect clinical disease persistence rather than detection of non-viable DNA [15].

Recent studies have shown that *M.genitalium* is capable of producing chronic infections in humans [18], and have focused on potential mechanisms of resistance. It appears that *M.genitalium* can undergo extensive gene sequence variation within a persistently infected individual [18], [19], [20], [21]. While mutations have been identified to explain the mechanism by which azithromycin resistance occurs [15], ongoing research indicates that resistance may develop *in vivo* after both single-dose and extended regimens of azithromycin (*personal communication*, *Jorgen Jensen*), suggesting that widespread use of 1 g of azithromycin may be contributing to the development of azithromycin-resistant *M.genitalium*.

There were some clear limitations to this study. This was a series of patients attending an STD clinic with specific indications for testing for *M.genitalium*. In this population, 11% of men and 10% of women selectively tested for *M.genitalium* were infected. These findings are relevant to symptomatic patients, but may not be generalisable to unselected, asymptomatic individuals. Only sixty-nine percent of patients testing positive for *M.genitalium* re-attended for toc1, compared >90% in our previous study; it is possible that men who re-attended were more likely to be experiencing persistent symptoms and infection compared to those lost to follow-up. Risk of reinfection relies on patient report, and may be unreliable. However, careful documentation of reinfection risk was undertaken in those with persistent *M.genitalium* due to attempts to limit inappropriate prescription of moxifloxacin.

The findings from this study highlight a number of important aspects in the diagnosis and treatment of *M.genitalium*. Mounting data suggest that while azithromycin 1 g is moderately effective, and superior to doxycyline, it is associated with unacceptable rates of disease persistence, and its widespread use could be selecting for resistance. *In vitro* data indicates that *M.genitalium* is highly sensitive to moxifloxacin[22], and it appears to be effective in individuals experiencing azithromycin failure; however, it is costly and there is a significant risk of the development of resistance if used inappropriately. As azithromycin is an effective, well-tolerated, single-dose therapy for NGU and cervicitis it is administered presumptively in many clinical settings, exposing a significant proportion of symptomatic patients with *M.genitalium* to azithromycin. This widespread practice may be selecting for azithromycin-resistance in *M.genitalium*. With studies now indicating a failure rate of at least 16%, follow-up of *M.genitalium*-infected patients prescribed azithromycin is not only becoming important, but further research is clearly needed to determine optimal first and second-line therapeutic agents for *M.genitalium*. This includes more rigorous evaluation of extended-azithromycin therapy, and moxifloxacin. The lack of access to a commercial assay for *M.genitalium* clearly poses a huge barrier for most clinical services in not only testing for this infection, but in assessing treatment efficacy and in monitoring the development of antibiotic resistance more broadly.
